# Weight Reduction via Lifestyle Intervention Improves Androgen Levels and Glucose Metabolism in Women of Reproductive Age with Hyperandrogenism: A Real-World Observational Study

**DOI:** 10.3390/jcm15124795

**Published:** 2026-06-20

**Authors:** Yang Yang, Zheng Liu, Jing Zhang

**Affiliations:** 1 Department of Obstetrics and Gynecology, West China Second University Hospital, Sichuan University, Chengdu 610041, China; yangyang0820@alu.scu.edu.cn (Y.Y.);; 2 Reproductive Endocrinology and Regulation Laboratory, West China Second University Hospital, Sichuan University, Chengdu 610041, China; 3 Key Laboratory of Birth Defects and Related Diseases of Women and Children, Ministry of Education, Sichuan University, Chengdu 610041, China; 4 The Joint Laboratory for Reproductive Medicine of Sichuan University, The Chinese University of Hong Kong, Chengdu 610041, China

**Keywords:** hyperandrogenism, lifestyle intervention, weight loss, glucose metabolism, androgen

## Abstract

**Background/Objectives**: Weight loss achieved through lifestyle interventions has been demonstrated to improve the clinical prognosis of female hyperandrogenism. However, the interplay between such interventions, androgens, and glucose–lipid metabolism remains heterogeneous. This study evaluated the effects of lifestyle-induced weight loss on glucose and lipid metabolism and androgen levels in Chinese women of reproductive age with hyperandrogenism and examined the association between the degree of weight loss and changes in androgen levels, glucose and lipid metabolism, exercise capacity, and dietary patterns. **Methods**: This observational study, based on real-world clinical settings, collected medical records of women of reproductive age with hyperandrogenism who underwent weight-loss interventions between July 2023 and September 2025. Correlation analysis employed Spearman’s rank correlation coefficient, whilst pre- and post-weight-loss comparisons utilised paired *t*-tests or Wilcoxon signed-rank tests. **Results**: After a follow-up of 6 to 7 months, a total of 66 participants achieved a mean weight loss of 5.67 ± 4.27 kg. Statistically significant reductions were observed in testosterone (0.40 ± 0.10 vs. 0.30 ± 0.10 ng/mL, *p* < 0.001), androstenedione (*p* < 0.001), and the free androgen index (*p* < 0.001). Glucose metabolism showed statistically significant improvement, with decreases in HOMA-IR (*p* = 0.040), fasting glucose (*p* = 0.001), and fasting/2 h postprandial insulin (*p* < 0.001). However, lipid profiles showed no statistically significant changes. Multiple linear regression revealed that change in testosterone was independently and inversely associated with change in apolipoprotein A1 (β = −0.496, *p* = 0.008), while change in dehydroepiandrosterone sulfate was inversely associated with change in fasting insulin (β = −0.357, *p* = 0.032). A non-linear, inverted U-shaped relationship was found between weight loss magnitude and change in sex hormone-binding globulin, with moderate weight loss (5–10%) yielding the greatest increase (*p* = 0.044). Marked weight loss (≥10%) was associated with the lowest follow-up fasting insulin levels (*p* = 0.039). **Conclusions**: Weight loss achieved through lifestyle interventions is associated with improvements in androgen levels and glucose metabolism, though its impact on lipid metabolism remains limited. The degree of improvement in insulin sensitivity correlates more strongly with the magnitude of weight reduction.

## 1. Introduction

Hyperandrogenism—characterised by elevated levels and/or heightened activity of one or more androgens in the circulation—is closely associated with disruption of the hypothalamic–pituitary–ovarian axis and energy metabolism in women; it may either result from or contribute to this disruption [1]. Hyperandrogenemia can be biochemical or clinical or both. It is frequently observed in polycystic ovary syndrome (PCOS) among women of reproductive age.

Previous clinical studies indicate a bidirectional, causal relationship between hyperandrogenism, obesity, and insulin resistance. Excess adipose tissue generates increased free fatty acids (FFA), reactive oxygen species (ROS), and pro-inflammatory cytokines, contributing to the development of insulin resistance. Many of these inflammatory factors directly stimulate ovarian androgen production while inhibiting the conversion of androgens to estrogens [2]. A state of hyperinsulinemia directly increases ovarian androgen synthesis by acting on the IGF-1 receptor and indirectly affects the liver, impairing the production of sex hormone-binding globulin (SHBG), ultimately leading to elevated circulating androgen levels [3].

The main causes of hyperandrogenemia in women include ovarian and adrenal overproduction of androgens, often driven by elevated luteinizing hormone (LH) and insulin, as well as decreased SHBG leading to increased free androgens. Additional contributing factors include obesity-related chronic inflammation, adipose tissue dysfunction, and genetic predisposition [4,5].

Weight management—encompassing weight loss, weight maintenance, or prevention of weight gain—constitutes the first-line foundational therapy for PCOS, achieved through interventions addressing diet, physical activity, and behavioural psychology. Lifestyle modification is the primary approach to weight management. Growing evidence suggests that compared with externally controlled interventions, self-determined interventions are more likely to produce lasting behavioural change, particularly in weight management and physical activity [6,7]. The role of weight loss in the correction of biochemical and hormonal anomalies is guideline-based in polycystic ovary syndrome.

The present study aimed to investigate the association between weight change, androgen levels, and glucose–lipid metabolism in reproductive-age women with hyperandrogenism undergoing self-directed weight loss.

## 2. Methods

### 2.1. Study Design

This study is a prospective, real-world observational study conducted in a routine clinical setting. The study protocol was approved by the Ethics Committee of West China Second University Hospital, Sichuan University (Approval No.: 2023-112; Approval Date: 25 June 2023). All participants signed a written informed consent form prior to enrolment after being fully informed of the study procedures.

### 2.2. Studied Population

Inclusion criteria: Eligible participants were women aged 18–40 years diagnosed with biochemical hyperandrogenism at the gynaecological endocrinology clinic, undergoing self-directed lifestyle modifications for weight management, and able to complete follow-up within 3 to 6 months. The diagnosis of hyperandrogenism adheres to the fundamental consensus on hyperandrogenism diagnosis outlined in the 2018 PCOS diagnostic criteria [8], supplemented by the specific clinical practices of our hospital’s laboratory department.

The intervention was based on self-determination theory, aiming to support participants’ autonomy and intrinsic motivation. No structured intervention manual was used; participants were given a single overall goal of “weight loss” without specific dietary or exercise prescriptions. At enrollment, each participant received standardised oral instructions from the researcher, including: a weight loss target of 0.5–1 kg per week [7], principles of fat reduction and muscle gain, and a list of knee-friendly exercise options (e.g., swimming, fixed resistance training) offered as suggestions rather than mandates. No other dietary or activity guidance was provided. The researcher acted as a facilitator rather than a director. Participants were followed weekly via a digital therapeutic platform. Each follow-up reiterated the weight loss goal and core recommendations and provided necessary health guidance or appointment reminders as needed. Structured behavioural recording was not required.

Exclusion criteria included: (i) Use of medications potentially affecting androgen levels or hyperandrogenism symptoms (e.g., spironolactone, isotretinoin, or short-term oral contraceptives) within 3 months prior to enrolment or during the study period; (ii) Identification during or prior to follow-up of organic causes of hyperandrogenism requiring surgical or pharmacological intervention, such as suspected adrenal or ovarian masses on CT or ultrasound, or suspected adrenal disease or ovarian neoplasms. (iii) Presence of severe medical or surgical comorbidities, or inability to adhere to the follow-up schedule.

Additional screening for adrenal androgens: For participants with markedly elevated androgen levels [9] (e.g., total testosterone > 2-fold the upper normal limit) accompanied by prominent clinical hyperandrogenism (e.g., severe hirsutism, virilization, clitoromegaly), clinicians performed additional tests including serum cortisol, ACTH, and 17-hydroxyprogesterone, and when indicated, adrenal imaging to exclude adrenal tumours or late-onset congenital adrenal hyperplasia. No routine dexamethasone suppression test was performed in all patients; such testing was reserved for those with specific clinical suspicion. The above exclusion criteria ensured that patients with confirmed organic causes requiring specific therapy were not included in the final analysis.

### 2.3. Study Protocol

This prospective observational study enrolled patients at the time of outpatient diagnosis. After providing written informed consent, participants underwent the self-directed lifestyle intervention described above, with weekly follow-up via a digital therapeutic platform. Physical measurements and blood samples were collected at baseline and during follow-up visits at 3 and 6 months, and assessments of nutritional status and physical fitness were conducted. Patients with irregular periods underwent a progesterone cycle to protect the endometrium. In accordance with clinical guidelines and ethical requirements, patients with oligomenorrhoea or amenorrhoea received short-term progestogen therapy (dydrogesterone or natural progesterone, for no more than 10 days) to induce withdrawal bleeding and protect the endometrium [10].

### 2.4. Outcomes

Blood samples for glucose and lipid metabolism tests were taken in the morning on an empty stomach. Blood samples for sex hormone testing were also taken in the morning; however, the day of the menstrual cycle was not recorded, nor were the samples taken at a specific phase of the cycle.

#### 2.4.1. Androgen Assessment

Venous blood samples (5 mL) were collected from participants at random time points, not restricted to a specific phase of the menstrual cycle. After collection, samples were placed in vacuum tubes containing a separation gel and allowed to clot at room temperature for 30 min. They were then centrifuged at 3000 rpm for 10 min to separate the serum. The isolated serum was aliquoted into 1.5 mL sterile microcentrifuge tubes and stored at −80 °C until analysis.

Serum levels of total testosterone (TT), androstenedione (AND), dehydroepiandrosterone sulfate (DHEAS), and SHBG were measured using Liquid Chromatography-Tandem Mass Spectrometry (LC-MS/MS). All procedures were performed in strict accordance with the manufacturer’s instructions for the respective reagent kits.

#### 2.4.2. Glucose Metabolism

Patients underwent a standard 75 g oral glucose tolerance test (OGTT) after an 8–12 h fast. Plasma glucose levels were measured using the glucose oxidase method at fasting and 2 h post-load (2 h PG). Simultaneously, serum insulin levels were assessed via chemiluminescent immunoassay at fasting (INS0) and 120 min (INS120) after glucose ingestion. The Homeostatic Model Assessment of Insulin Resistance (HOMA-IR) was calculated from the fasting measurements.

#### 2.4.3. Lipid Metabolism

Fasting blood samples were obtained for lipid profile analysis. Total cholesterol (TC) and triglycerides (TG) were measured using the glycerol phosphate oxidase-p-aminophenol method. Lipoprotein electrophoresis was employed to quantify low-density lipoprotein cholesterol (LDL-C), high-density lipoprotein cholesterol (HDL-C), apolipoprotein A (Apo A), and apolipoprotein B (Apo B). Homocysteine (HCY) levels were determined using an enzymatic cycling assay.

#### 2.4.4. Exercise Capacity and Nutritional Patterns

A muscle function analyzer (MES-01S20; Beijing MDK Medical Equipment Manufacturing Co., Ltd., Beijing, China) was utilised for objective physical assessments. This non-radioactive, non-invasive device measured total body fat percentage, lower limb muscle distribution, lower limb muscle strength, knee joint range of motion, static and dynamic balance, and metabolic rate. Additionally, a dedicated sports nutrition professional conducted one-on-one evaluations for each patient, assessing body composition (fat and muscle distribution), physical function, metabolic status, and dietary nutritional intake.

### 2.5. Statistical Analysis

All data organisation and statistical analyses were performed using IBM SPSS Statistics (version 26.0). Continuous variables were tested for normality using the Shapiro–Wilk test; normally distributed data are presented as mean ± standard deviation (SD), and non-normally distributed data as median (interquartile range, P25–P75). Baseline characteristics, follow-up durations, and changes (Δ) in hormonal and metabolic parameters across the three strata were compared using one-way analysis of variance (ANOVA; F-value reported) or the Kruskal–Wallis test (χ^2^-value reported), with post hoc tests where appropriate. Multiple linear regression analyses were performed to examine independent associations: Model 1 assessed the relationship between changes in androgens (ΔT, ΔDHEAS) and changes in metabolic markers (ΔApo A1, ΔINS0), adjusting for age and ΔBMI; Model 2 evaluated the association between ΔBMI and ΔHDL-C, adjusting for age and ΔT. Standardised beta coefficients (β) and corresponding *p*-values are reported. No imputation methods were used; only valid cases were analysed. To assess potential bias from missing data, baseline characteristics of participants who completed the intervention were compared with those who did not, using an independent-samples *t*-test (normally distributed variables) or the Mann–Whitney U test (non-normally distributed variables). This was a real-world observational study; sample size was determined by consecutive eligible cases during the study period, without a priori power calculation. The primary outcome was change in serum total testosterone (T). Post hoc power analysis using G*Power 3.1 (α = 0.05, two-tailed; observed effect size Cohen’s d = 0.83, *n* = 46 for those with sex hormone measurements) yielded a power of 0.99, indicating sufficient power to detect the observed change in testosterone. However, for a medium effect size (d = 0.5, *n* = 46), power was approximately 0.68, and for a small effect size (d = 0.2), power was <0.30. Given the exploratory nature of this study, no strict α adjustment was made for multiple comparisons. A total of approximately 35 hypothesis tests (including group comparisons, pre-post comparisons, subgroup analyses, and regression models) were performed. For primary outcomes (testosterone, fasting glucose, HOMA-IR), *p* < 0.05 was considered statistically significant; for exploratory secondary outcomes, *p* < 0.05 served only as a signal for hypothesis generation, requiring confirmation in future studies.

## 3. Results

**Figure 1:**
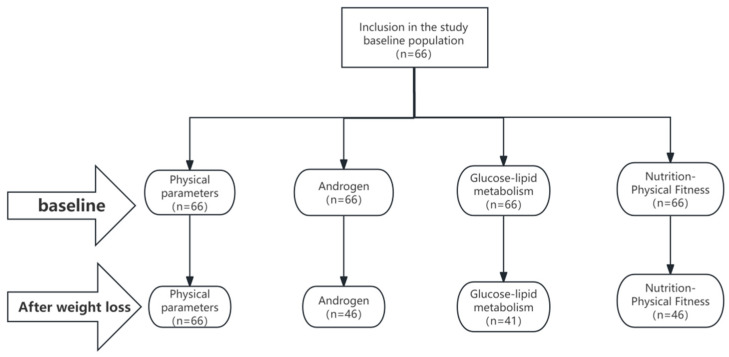
Flowchart of research sample variables.

Of the 66 participants enrolled, 5 (7%) completed all scheduled follow-ups. Following weight loss, physical parameters (such as weight and BMI) were measured for all 66 participants. Forty-six participants underwent serum androgen testing and nutritional and physical fitness assessments, whilst glucose and lipid metabolism data were available for analysis in 41 participants (Figure 1). Regarding weekly follow-up: in the first month, 65% (43/66) of participants completed at least three of the up to four monthly follow-up contacts; in the second month, 28% (19/66) of participants completed at least three contacts. A total of 20 participants declined post-intervention serological and exercise/nutritional assessments. Reasons given included feeling well after weight loss and the return of normal menstruation (*n* = 12), as well as conflicts with work schedules (*n* = 8).

Of the 66 participants, 46 (69.7%) completed the post-intervention androgen test (the completion group), whilst 20 (30.3%) did not (the non-completion group). To assess whether missing data might introduce selection bias, we compared the baseline characteristics of the two groups (Table 1). The results showed no significant differences between the completion group and the non-completion group across all baseline variables (all *p* > 0.05), suggesting that the missing data from the post-intervention androgen testing did not introduce significant bias due to these measured variables.

A total of 69% (46/66) of patients underwent two rounds of sex hormone testing. Results showed significant decreases in testosterone, androstenedione, and free androgen index levels in all subjects after follow-up (*p* < 0.001), as presented in Table 2.

A total of 62% (41/66) of patients demonstrated significant reductions in insulin resistance index, fasting blood glucose, fasting insulin, 120 min insulin, and 120 min blood glucose both before and after follow-up (*p* < 0.05). However, lipid metabolism-related indicators showed no significant differences before and after intervention (*p* > 0.05), as shown in Table 3.

Upon follow-up, 69% (46/66) of the cohort showed alterations in physical measurements and nutritional parameters, as detailed in Table 4. A reduction in total body fat percentage was observed (mean difference −3.817%, 95% CI: −4.841, −2.793, *p* < 0.001), and basal metabolic rate also decreased (mean difference −45.321 kcal/day, 95% CI: −60.619, −30.022, *p* < 0.001). In contrast, the effective range of motion of the knee joint did not change significantly (mean difference −0.981°, 95% CI: −6.995, 5.033, *p* = 0.745). Regarding dietary habits, increases were observed in daily intake of dietary fibre (mean difference 4.017 g, 95% CI: 0.378, 7.656, *p* = 0.031), vitamin E (5.877 mg, 95% CI: 0.376, 11.378, *p* = 0.037), phosphorus (86.196 mg, 95% CI: 4.048, 168.343, *p* = 0.020), iodine (9.059 μg, 95% CI: 3.857, 14.261, *p* = 0.001), and pantothenic acid (1.320 mg, 95% CI: 0.066, 2.573, *p* = 0.039), as well as the percentage of energy derived from protein (1.009%, 95% CI: 0.050, 1.967, *p* = 0.040). Daily sodium intake was reduced (mean difference −615.100 mg, 95% CI: −1224.939, −5.260, *p* = 0.048).

To investigate the dose–response relationship of weight reduction, patients were stratified into three groups based on the percentage of initial body weight lost, in accordance with established criteria [4]: mild weight loss (<5%), moderate weight loss (5% to <10%), and marked weight loss (≥10%). The median follow-up duration was 6–7 months, with no significant differences in follow-up time observed among the three groups (*p* = 0.158). Furthermore, baseline characteristics—including age, weight, BMI, waist circumference, hip circumference, and waist-to-hip ratio—were comparable across all groups (*p* > 0.05).

Multiple linear regression analyses were conducted to identify independent associations between changes in hormonal profiles and metabolic indicators, adjusting for age and changes in BMI (ΔBMI) (Table 5a). These models suggested an independent inverse association between the change in total testosterone (ΔT) and the change in apolipoprotein A1 (ΔApo A1) (standardised β = −0.496, 95% CI for β: −1.941, −0.558, *p* = 0.008). Similarly, the change in dehydroepiandrosterone sulfate (ΔDHEAS) was inversely associated with the change in fasting insulin (ΔINS0) (standardised β = −0.357, 95% CI for β: −0.068, −0.003, *p* = 0.032). In separate models adjusting for age and ΔT (Table 5b), the change in BMI (ΔBMI) was associated with the change in high-density lipoprotein cholesterol (ΔHDL-C) (standardised β = −0.451, 95% CI for β: −1.439, −0.059, *p* = 0.044).

Changes in sex hormone-binding globulin (ΔSHBG) differed across the three weight-loss strata (*p* = 0.044, Table 6). The moderate weight loss group showed a median ΔSHBG of 5.40 nmol/L, compared with −0.70 nmol/L in the mild loss group and 2.90 nmol/L in the marked loss group. No significant differences were observed across the groups for changes in total testosterone (ΔT), DHEAS, FAI, or androstenedione (ΔAND) (all *p* > 0.05).

Most metabolic parameters at the end of follow-up were similar across the three weight-loss strata (Table 7). A difference was observed in fasting insulin levels (INS0) (*p* = 0.039): the marked weight loss group had the lowest median INS0 (9.61 µIU/mL), whereas the mild loss group had the highest (15.81 µIU/mL). The findings for HOMA-IR and other lipid parameters remained inconclusive.

## 4. Discussion

In this real-world observational study of women of childbearing age with hyperandrogenism, we observed that weight loss achieved through an autonomous lifestyle intervention programme based on self-determination theory significantly reduced serum testosterone, androstenedione and the free androgen index whilst also improving fasting blood glucose, fasting insulin, HOMA-IR, 2 h postprandial glucose levels, body fat percentage, basal metabolic rate and the intake of various dietary nutrients. In contrast, lipid parameters such as total cholesterol, triglycerides, HDL-C, LDL-C and apolipoproteins did not show statistically significant changes. These findings suggest that, for this population, a self-determination theory-based autonomous lifestyle intervention programme may be a viable strategy for improving hyperandrogenism and glucose metabolism; however, its impact on the lipid profile remains inconclusive.

Our findings are consistent with the existing consensus that weight loss reduces adipose tissue-mediated insulin resistance and the resulting ovarian androgen production, which is a key pathophysiological mechanism underlying the control of hyperandrogenism through weight management [11,12]. The reductions in testosterone and the free androgen index observed in our study are consistent with the findings of a recent systematic review and network meta-analysis of 21 randomised controlled trials (1196 participants) [13], which concluded that non-pharmacological interventions (such as diet, exercise and lifestyle changes) can significantly reduce serum testosterone levels in women with PCOS. Furthermore, the reduction in AND levels observed in this study (–0.918 ng/mL, 95% CI: −1.340 to −0.496, *p* < 0.001) is consistent with the findings of a network meta-analysis, which indicated that non-pharmacological interventions significantly reduce androstenedione levels. This suggests that the real-world results of this study are generally consistent with those observed in more controlled experimental settings in terms of both the direction and magnitude of the effect.

With regard to glucose metabolism, the improvements in fasting blood glucose, fasting insulin, HOMA-IR and 2 h postprandial blood glucose observed in this cohort are consistent with the findings of a large systematic review encompassing 29 randomised controlled trials, which found that weight-loss interventions were associated with significant improvements in HOMA-IR (mean difference −0.45, 95% CI: −0.75 to −0.15) and fasting insulin levels [14].

Our study observed no significant changes in lipid parameters (TC, TG, LDL-C, HDL-C) following weight loss; this may be due to the study’s insufficient sample size to detect small to moderate changes in lipid parameters and the short duration of the intervention. We observed an independent negative correlation between changes in testosterone and Apo A1 (ΔApo A1; β = −0.496, 95% CI: −1.941, −0.558, *p* = 0.008), suggesting that as testosterone levels decrease with weight loss, Apo A1—a key component of HDL particles—may consequently increase. Furthermore, the independent association between reduced BMI and improved HDL-C levels (β = −0.451, 95% CI: −1.439, −0.059, *p* = 0.044) suggests that weight loss itself—rather than merely a reduction in androgen levels—may directly influence the lipid profile. A 2026 systematic review and network meta-analysis incorporating 23 randomised controlled trials found that exercise had no significant effect on HDL cholesterol, triglycerides or blood glucose levels, suggesting that the impact of exercise on improving the lipid profile in patients with polycystic ovary syndrome varies and may depend on the type of intervention and the extent of weight loss [15].

We also observed a negative correlation between DHEAS and fasting insulin levels in our regression analysis (β = −0.357, 95% CI: −0.068, −0.003, *p* = 0.032). We speculate that the negative correlation between ΔDHEAS and fasting insulin in this study may reflect a potential protective role of DHEAS in insulin metabolism—a hypothesis consistent with findings from neuroendocrinological studies [16], which indicate that DHEAS may exert a neuroprotective effect in women with PCOS by mitigating age-related changes in the brain [17,18]. Hyperandrogenism may influence cardiovascular risk through multiple pathways, some of which may be protective, whilst others may have adverse effects [19,20].

A striking finding was the non-linear relationship between the extent of weight loss and changes in SHBG. Moderate weight loss (5–10%) resulted in the most significant increase in SHBG (median Δ = 5.40 nmol/L, *p* = 0.044), whereas mild (<5%) and substantial (≥10%) weight loss showed smaller increases. We hypothesise that weight loss alleviates the inhibitory effect of hyperinsulinaemia on hepatic SHBG synthesis whilst avoiding the metabolic burden on the liver associated with very low calorie intake or excessive weight loss; this inverted U-shaped pattern suggests that moderate weight loss most effectively stimulates hepatic SHBG production [21,22]. The plateau or decline in the SHBG response following significant weight loss may reflect compensatory mechanisms or the metabolic consequences of substantial calorie restriction. However, it should be emphasised that the sample size was limited after stratification (significant weight loss group, *n* = 16), and this non-linear pattern remains to be validated and confirmed by prospective studies with larger sample sizes.

With regard to glucose metabolism, fasting insulin exhibited a more pronounced dose–response pattern: the fasting insulin levels were the lowest in the significant weight loss group (median 9.61 µIU/mL), and there was a statistically significant difference compared with the mild weight loss group (median 15.81 µIU/mL). This is consistent with the prevailing view in the existing literature that greater weight loss is associated with more significant improvements in insulin sensitivity [23]. However, it must be acknowledged that although HOMA-IR is a comprehensive indicator of insulin resistance, HOMA-IR values did not show significant differences between the different weight loss strata in this study—this inconsistency may be attributed to the relatively small sample sizes in each subgroup.

The significant decrease in resting metabolic rate following weight loss (1377.98 vs. 1330.09 kcal/day, *p* < 0.001) reflects the body’s anticipated physiological adaptation to weight loss, reflecting both a reduction in fat mass and a possible loss of metabolically active lean tissue. This metabolic adaptation poses a challenge to weight maintenance and highlights the importance of preserving muscle mass during weight loss interventions. A recent meta-analysis involving 57 studies and 2963 participants further confirms that reducing carbohydrate intake during a low-calorie diet may help maintain the post-weight-loss basal metabolic rate, particularly for individuals who have lost more than 5% of their body weight [24]. It is worth noting that the decline in basal metabolic rate observed in this study is consistent with the conclusions of a large narrative review from 2024 [25], which indicated that the inhibitory effects of weight loss on resting energy expenditure and adaptive thermogenesis are among the most significant physiological mechanisms contributing to the extremely high relapse rates (>80%) in weight management.

No significant improvements were observed in knee range of motion, dynamic balance, static balance, or muscle strength, suggesting that weight loss alone, without systematic resistance training, may be insufficient to adequately maintain or enhance physical function [26,27]. A systematic review suggested that resistance training during diet-induced weight loss can prevent lean body mass loss and may improve muscle function [28]. A 2025 pilot randomised controlled trial further confirmed that incorporating home-based resistance training into a weight-loss programme can maintain or even enhance muscle function without adversely affecting the outcomes of dietary weight loss [29]. Consistent with this, a 2025 review on strategies for maintaining and increasing muscle mass during fat loss suggested that resistance training is a key component of maintaining lean body mass during calorie restriction [30]. Therefore, we recommend that significant improvements in neuromuscular and functional outcomes for this population may require the deliberate incorporation of balance and strength training elements, rather than relying solely on weight loss itself.

Dietary assessments following the intervention revealed a significant increase in the intake of various micronutrients, including dietary fibre, vitamin E, phosphorus, iodine and pantothenic acid, alongside a reduction in sodium intake and an increase in the proportion of protein in total energy intake. A 2026 narrative review on nutrients in the management of PCOS noted that vitamin E has multifaceted potential in alleviating insulin resistance and improving endocrine imbalances in PCOS; its mechanisms of action may include activating antioxidant regulatory mechanisms, mitigating chronic inflammatory responses, and reducing lipid peroxidation and lipid synthesis. Vitamin E supplementation can effectively lower levels of TT, luteinising hormone (LH) and LDL-C levels [31]. Another study found that dietary fibre intervention can improve glycaemic control, insulin sensitivity and weight management in overweight or obese individuals and that overall dietary fibre intake can lower fasting blood glucose levels [32]. However, further research is needed to determine whether it is these improvements in nutritional status that promote weight loss or whether the process of weight loss itself leads individuals to choose healthier foods. It is highly likely that there is a bidirectional feedback loop between weight loss and changes in dietary behaviour.

The increase in pantothenic acid (vitamin B5) intake is noteworthy, as B vitamins play a key role as cofactors in the tricarboxylic acid cycle, amino acid metabolism and lipid metabolism [33,34]. Adequate B vitamin levels may support metabolic flexibility during weight loss and help maintain oxidative capacity despite reduced calorie intake [35]. However, it must be noted that this study did not measure baseline vitamin B levels, nor did it assess changes in downstream markers such as acetyl-CoA intermediates or citric acid cycle flux; therefore, these mechanistic explanations remain speculative.

This real-world, patient-led weight management practice has revealed that moderate weight loss (5–10%) constitutes a feasible and metabolically beneficial target, potentially offering the greatest potential for improving sex hormone-binding globulin levels and reducing androgen levels [36]. During sustained weight loss, fasting insulin levels serve as a sensitive indicator of metabolic improvement. Such weight management is associated with reduced metabolic rate and no increase in strength; therefore, incorporating resistance training into weight loss programmes is crucial for preserving lean body mass and metabolic capacity.

We must acknowledge that this study has certain limitations. Firstly, as a real-world observational study designed to support autonomy and intrinsic motivation through an intervention based on Self-Determination Theory (SDT), we did not collect daily behavioural logs, such as records of dietary intake or physical activity. Consequently, we are unable to report quantitative measures of adherence. The non-compulsory, autonomy-oriented nature of the intervention; the high early dropout rate we observed; and the fact that some participants declined to continue follow-up because they ‘felt better’ or their ‘menstrual cycles had resumed’ suggest that, for certain individuals, the intervention successfully internalised health goals, leading them to no longer consider participation in the study follow-up necessary for their personal health journey. This reflects the potential for individual variation in the tendency towards autonomous internalisation. Previous studies suggest that for patients receiving interventions based on Self-Determination Theory, early identification of low responders is necessary to enhance intervention effectiveness and provide timely, tailored interventions [37,38]. Therefore, in future clinical practice, attempts could be made to assess patients’ levels of intrinsic motivation and readiness for self-management prior to intervention in order to provide personalised interventions.

Secondly, the timing of sex hormone sampling in this study was not standardised to a specific phase of the menstrual cycle. Although glucose and lipid parameters were measured following an overnight fast, and all androgen samples were collected in the morning, the specific date of the menstrual cycle was not recorded; we acknowledge this as a limitation of the study. The 2024 International Evidence-Based Guidelines Systematic Review still recommends the use of serum total testosterone levels in the early follicular phase where feasible [39]. In clinical practice, it is challenging to define the ‘early follicular phase’ before measuring androgens in patients with suspected PCOS, who often present with oligomenorrhoea or amenorrhoea.

Finally, this study had a small sample size (*n* = 66) and no prior sample size estimation was performed, resulting in limited statistical power. Post hoc power analyses indicated that the study had adequate power (≥0.68) for moderate or larger effect sizes but insufficient power for smaller effects, making it prone to false-negative results. Therefore, the outcomes for which no significant differences were observed in this study require validation in studies with larger sample sizes.

A significant proportion of participants in this study voluntarily discontinued follow-up due to ‘feeling better’ or ‘return of menstruation’, a phenomenon of considerable clinical significance. Firstly, in real-world settings, a patient’s decision to withdraw from structured follow-up may indicate the success of the intervention rather than its failure. Clinicians should recognise that such ‘voluntary withdrawal’ may reflect successful self-management. Secondly, individuals with low self-determination should be identified at an early stage so that support for follow-up can be strengthened on an individualised basis. Finally, patient self-satisfaction and improved quality of life are equally important as improvements in biological endpoints. Future research should formally validate whether interventions based on Self-Determination Theory can yield better long-term adherence and behavioural maintenance outcomes than traditional directive approaches.

## 5. Conclusions

Based on the findings of this real-world observational study, we propose the following conclusions, which are preliminary and require further validation: 1. Moderate weight loss (5–10%) achieved through lifestyle interventions based on self-determination theory and self-efficacy can significantly improve hyperandrogenism (reduced testosterone, androstenedione and free androgen index) and glucose metabolism (reduced fasting blood glucose, insulin and HOMA-IR), with the most significant increase in sex hormone-binding globulin observed in the moderate weight loss subgroup. However, given the limited sample size, these results should be interpreted with caution. 2. Weight loss alone, without structured resistance training, fails to improve muscle strength and dynamic or static balance or increase knee range of motion; therefore, resistance training should be incorporated into weight management programmes to maintain lean body mass and metabolic capacity. 3. The decline in resting metabolic rate observed following weight loss reflects physiological adaptive mechanisms and poses a significant challenge to weight maintenance; strategies such as adequate protein intake and resistance training are crucial for mitigating this effect. However, direct evidence is currently lacking. 4. Improvements in nutritional status (increased dietary fibre, vitamin E, pantothenic acid and protein intake) were observed during weight loss, but the causal relationship remains unclear; nevertheless, attention to dietary quality should still be emphasised in clinical practice. 5. The attrition of study participants may stem from positive factors, suggesting that self-determined interventions may successfully promote the internalisation of healthy behaviours; to improve adherence, it may be necessary to identify individuals with low motivation at an early stage. Future research should focus on optimising intervention strategies and assessing long-term sustainability, thereby translating these findings into more effective and personalised clinical practice.

## Figures and Tables

**Table 1:** Baseline characteristics of completers vs. non-completers of post-intervention androgen testing.

Parameter	Completers (*n* = 46)	Non-Completers (*n* = 20)	Mean Difference (95% CI)	T/Z	*p*
Age (years)	25.00 (22.00, 26.50)	25.58 ± 4.52	−1.00 (−3.00, 1.00)	1.205	0.305
BMI (kg/m^2^)	26.86 ± 3.80	28.61 ± 3.68	−1.82 (−3.74, 0.10)	−1.890	0.063
T (ng/mL)	0.40 ± 0.10	0.40 ± 0.10	−0.01 (−0.06,0.04)	−0.403	0.688
FBG (mmol/L)	4.93 (4.74, 5.55)	5.11 ± 0.51	0.00 (−0.29, 0.26)	0.049	0.961

Mann–Whitney U test for age and FBG (Z); *t*-test for BMI and T (T).

**Table 2:** Paired-sample *t*-test of sex hormone indicators before and after weight loss in study subjects (*n* = 46).

Parameter	Before Weight Loss (Mean ± SD or M (P25, P75))	After Weight Loss (Mean ± SD or M (P25, P75))	Mean Difference (95% CI)	*p*
T (ng/mL)	0.40 ± 0.10	0.30 ± 0.10	−0.088 (−0.116, −0.060)	<0.001
AND (ng/mL)	4.37 ± 1.46	3.37 ± 1.52	−0.918(−1.340, −0.496)	<0.001
DHEAS (µg/dL)	287.34 ± 94.87	304.92 ± 116.22	3.568(−23.255, 30.392)	0.790
SHBG (nmol/L)	19.30 (13.70, 35.00)	25.95 (15.40, 38.67)	0.943 (−3.571, 5.457)	0.676
FAI (%)	6.55 (4.52, 10.85)	3.60 (2.57, 7.62)	−2.166(−3.306, −1.026)	<0.001

**Table 3:** Paired-sample *t*-test of glucose metabolism indicators before and after intervention in study subjects (*n* = 41).

Parameter	Before Weight Loss (Mean ± SD or M (P25, P75))	After Weight Loss (Mean ± SD or M (P25, P75))	Mean Difference (95% CI)	*p*
HOMA-IR	4.40 ± 2.00	3.53(2.58, 4.20)	−2.076 (−2.822, −1.330)	0.040
FPG (mmol/L)	5.10 ± 0.49	4.86 ± 0.24	−0.222 (−0.350, −0.095)	0.001
2 h PG (mmol/L)	7.76 ± 1.88	4.96 ± 1.39	−0.947 (−1.698, −0.197)	0.016
INS0 (µIU/mL)	18.33 (13.82, 28.60)	16.81 ± 5.99	−7.858 (−10.598, −5.117)	<0.001
INS120 (µIU/mL)	135.93 ± 99.06	108.81 ± 113.43	−83.132 (−118.426, −47.837)	<0.001
TC (mmol/L)	4.49 ± 1.01	4.46 ± 0.63	−0.025 (−0.350, 0.301)	0.878
TG (mmol/L)	1.16 (0.97, 1.76)	1.05 ± 0.39	−0.313 (−1.389, 0.763)	0.555
HDL-C (mmol/L)	1.18 ± 0.24	1.30 ± 0.13	0.368 (−0.375, 1.110)	0.319
HCY (µmol/L)	8.87 (7.99, 10.6)	10.21 ± 4.02	−0.619 (−1.866, 0.628)	0.318
LDL-C (mmol/L)	2.97 ± 0.95	3.2 ± 1.25	0.080 (−0.194, 0.355)	0.553
Apo A1 (g/L)	1.27 ± 0.15	1.30 ± 0.14	0.061 (−0.009, 0.131)	0.083
Apo B (g/L)	0.77 (0.65, 0.92)	0.84 ± 0.27	0.007 (−0.038, 0.053)	0.750

**Table 4:** Changes in physical function and nutrient intake before and after weight loss (*n* = 46).

Parameter	Before Weight Loss (Mean ± SD or M (P25, P75))	After Weight Loss (Mean ± SD or M (P25, P75))	Mean Difference (95% CI)	*p*
Knee Joint Effective Range of Motion (°)	129.36 ± 15.135	128.38 ± 24.299	−0.981 (−6.995, 5.033)	0.745
Total Body Fat Percentage (%)	40.87 ± 7.13	36.86 ± 7.13	−3.817 (−4.841, −2.793)	<0.001
Metabolic Rate (kcal/day)	1377.98 ± 107.42	1330.09 ± 118.97	−45.321 (−60.619, −30.022)	<0.001
Dietary Fibre Daily Intake (g)	22.23 (18.66, 28.29)	25.67 (20.54, 33.58)	4.017 (0.378, 7.656)	0.031
Vitamin E Daily Intake (mg)	32.46 (24.49, 45.28)	37.94 (29.12, 56.48)	5.877 (0.376, 11.378)	0.037
Phosphorus Daily Intake (mg)	1160.88 ± 207.12	1196.5 (1043.68, 1433.50)	86.196 (4.048, 168.343)	0.020
Sodium Daily Intake (mg)	1929.44 (1358.86, 2986.61)	1582.0 (1195.00, 2122.56)	−615.100 (−1224.939, −5.260)	0.048
Iodine Daily Intake (μg)	37.68 (30.07, 46.01)	45.20 (36.72, 60.77)	9.059 (3.857, 14.261)	0.001
Pantothenic Acid Daily Intake (mg)	7.66 (6.61, 9.61)	8.62 (6.92, 11.25)	1.320 (0.066, 2.573)	0.039
Protein Contribution to Energy Intake (%)	16.36 ± 2.82	17.44 ± 2.33	1.009 (0.050, 1.967)	0.040

**Table 5:** (**a**) Independent associations of changes in androgens with apolipoprotein and insulin metabolism. (**b**) Independent association of weight loss magnitude with HDL-cholesterol improvement.

(**a**)				
**Dependent Variable (Δ)**	**Independent Variable (Δ)**	**β (Std.)**	**95% CI for β**	***p***
Apo A1 (g/L)	T (ng/mL)	−0.496	(−1.941, −0.558)	0.008
INS0 (µIU/mL)	DHEAS (µg/dL)	−0.357	(−0.068, −0.003)	0.032
(**b**)				
**Dependent Variable (Δ)**	**Independent Variable (Δ)**	**β (Std.)**	**95% CI for β**	***p***
HDL-C (mmol/L)	BMI (kg/m^2^)	−0.451	(−1.439, −0.059)	0.044

(a) Models adjusted for age and ΔBMI; (b) Models adjusted for age and ΔT.

**Table 6:** Changes in androgen-related profiles across weight-loss strata.

Variable	Degree of Weight Reduction (Mean ± SD)			F/X^2^	*p*
	Mild (*n* = 26)	Moderate (*n* = 24)	Marked (*n* = 16)		
ΔT	−0.06 ± 0.08	−0.09 ± 0.10	−0.13 ± 0.05	1.889	0.164
ΔAND	−0.410 ± 1.06	−1.31 ± 1.53	−1.32 ± 1.52	2.366	0.107
ΔDHEAS	3.39 ± 83.70	5.73 ± 91.02	1.00 ± 105.12	0.008	0.992
ΔSHBG	−0.70 (−2.62, 0.80)	5.40 (0.07, 7.40)	2.90 (−0.82, 11.70)	6.249	0.044
ΔFAI	−0.90 ± 2.15	−2.64 ± 2.86	−3.9 4 ± 6.16	2.361	0.107

Due to variations in the normality of the dependent variables, two statistical methods were employed to compare differences among the three groups: analysis of variance (F-value) and the non-parametric Kruskal–Wallis test (X^2^-value).

**Table 7:** Changes in Glucose–Lipid Metabolism-Related Indicators Across Different Weight Loss Levels.

Project at Follow-Up	Degree of Weight Reduction (Mean ± SD)			F/X^2^	*p*
	Mild (*n* = 17)	Moderate (*n* = 14)	Marked (*n* = 10)		
HOMA-IR	4.18 ± 3.61	3.06 ± 1.20	3.68 ± 0.14	0.281	0.759
FBG (mmol/L)	5.04 ± 0.31	4.96 ± 0.45	4.81 ± 0.41	1.168	0.321
GLU2 (mmol/L)	6.72 ± 1.25	9.54 ± 0.38	6.70 ± 0.75	0.693	0.510
INS0 (µIU/mL)	15.81 (11.55, 22.43)	12.05 (10.46, 18.70)	9.61 (6.59, 13.22)	6.481	0.039
INS120 (µIU/mL)	65.69 (50.85, 101.33)	96.61 (64.45, 105.85)	50.60 (35.91, 95.35)	1.926	0.382
TC (mmol/L)	4.46 ± 0.74	4.32 ± 0.73	4.24 ± 0.75	0.264	0.770
TG (mmol/L)	1.30 (1.12, 2.45)	1.13 (0.84, 3.14)	0.97 (0.84, 1.56)	1.387	0.265
HDL-C (mmol/L)	1.12 ± 0.24	1.18 ± 0.29	1.16 ± 0.24	0.202	0.818
LDL-C (mmol/L)	2.96 ± 0.71	2.66 ± 0.87	2.85 ± 0.97	0.397	0.676
HCY (umol/L)	9.32 ± 2.53	8.45 ± 1.89	10.01 ± 3.58	0.860	0.433

## Data Availability

The raw data supporting the conclusions of this manuscript will be made available by the authors, without undue reservation, to any qualified researcher.

## Abbreviations

The following abbreviations are used in this manuscript:

FFA	Free fatty acids
ROS	Reactive oxygen species
IGF-1	Insulin-like Growth Factor 1
SHBG	Sex hormone-binding globulin
LH	Luteinizing hormone
PCOS	Polycystic Ovary Syndrome
ACTH	Adrenocorticotropic hormone
TT	Total testosterone
AND	Androstenedione
DHEAS	Dehydroepiandrosterone sulfate
LC-MS/MS	Liquid Chromatography-Tandem Mass Spectrometry
OGTT	Oral glucose tolerance test
INS0	Insulin at 0 min (fasting insulin)
INS120	Insulin at 120 min
FBG	Fasting blood glucose
2 h PG	2 h plasma glucose
TC	Total cholesterol
TG	Triglycerides
LDL-C	Low-density lipoprotein cholesterol
HDL-C	High-density lipoprotein cholesterol
Apo A1	Apolipoprotein A1
Apo B	apolipoprotein B
HCY	Homocysteine
BMI	Body mass index
HOMA-IR	Homeostatic Model Assessment for Insulin Resistance
SDT	Self-Determination Theory
